# Pyrimethamine inhibits cell growth by inducing cell senescence and boosting CD8^+^ T-cell mediated cytotoxicity in colorectal cancer

**DOI:** 10.1007/s11033-022-07262-y

**Published:** 2022-03-09

**Authors:** Haiyan Dong, Limei Hu, Weiqian Li, Mengchen Shi, Lingyuan He, Chen Wang, Yijia Hu, Huihui Wang, Chuangyu Wen, Huanliang Liu, Xiangling Yang

**Affiliations:** 1 Department of Clinical Laboratory, The Sixth Affiliated Hospital, Sun Yat-Sen University, Guangzhou, Guangdong China; 2 Guangdong Provincial Key Laboratory of Colorectal and Pelvic Floor Diseases, Guangdong Institute of Gastroenterology, The Sixth Affiliated Hospital, Sun Yat-Sen University, Guangzhou, Guangdong China; 3 Department of Obstetrics and Gynecology, Affiliated Dongguan Hospital, Southern Medical University, Dongguan, Guangdong China

**Keywords:** Pyrimethamine, Colorectal cancer, Cellular senescence, p38MAPK, p53, CD8^+^ T cell

## Abstract

**Background:**

The emergence of nonresponse or resistance to traditional chemotherapeutic agents is one of the main challenges of colorectal cancer (CRC) therapies. Thus, novel therapeutic drugs that can improve the clinical outcomes of CRC patients are urgently needed. The purpose of this study was to investigate the effects and mechanisms of pyrimethamine in CRC.

**Methods and results:**

In this study, we assessed the role of pyrimethamine on CRC cell growth by cell counting kit-8 and colony formation assays. Cell cycle distribution and cellular senescence were determined by flow cytometry and senescence-associated β-galactosidase staining respectively. RNA-seq analysis and western blotting were used to investigate the potential pathways of pyrimethamine in CRC development. Moreover, animal experiments were performed to evaluate the effect of pyrimethamine in vivo. Our results demonstrated that pyrimethamine could inhibit cell growth by inducing S phase arrest followed by cellular senescence in CRC cells, and the p38MAPK-p53 axis was probably involved in that effect. In addition, pyrimethamine could also boost CD8^+^ T-cell mediated cytotoxicity and exert antitumor activity in vivo.

**Conclusion:**

These results indicated that pyrimethamine may be a promising candidate agent for CRC treatment.

## Introduction

Colorectal cancer (CRC) is the third most common cancer and accounts for the second leading cause of cancer-related mortality worldwide [1]. Surgery and chemotherapy are the main treatment options for CRC. Nevertheless, the therapeutic effect of traditional chemotherapy drugs is not always satisfactory. For instance, nearly 50% of metastatic CRC patients are resistant to 5-fluorouracil-based chemotherapies, which limits their efficacy and results in treatment failure [2]. Hence, it is urgently needed to explore novel therapeutic drugs that can improve the clinical outcomes of CRC patients.

**Fig. 1:**
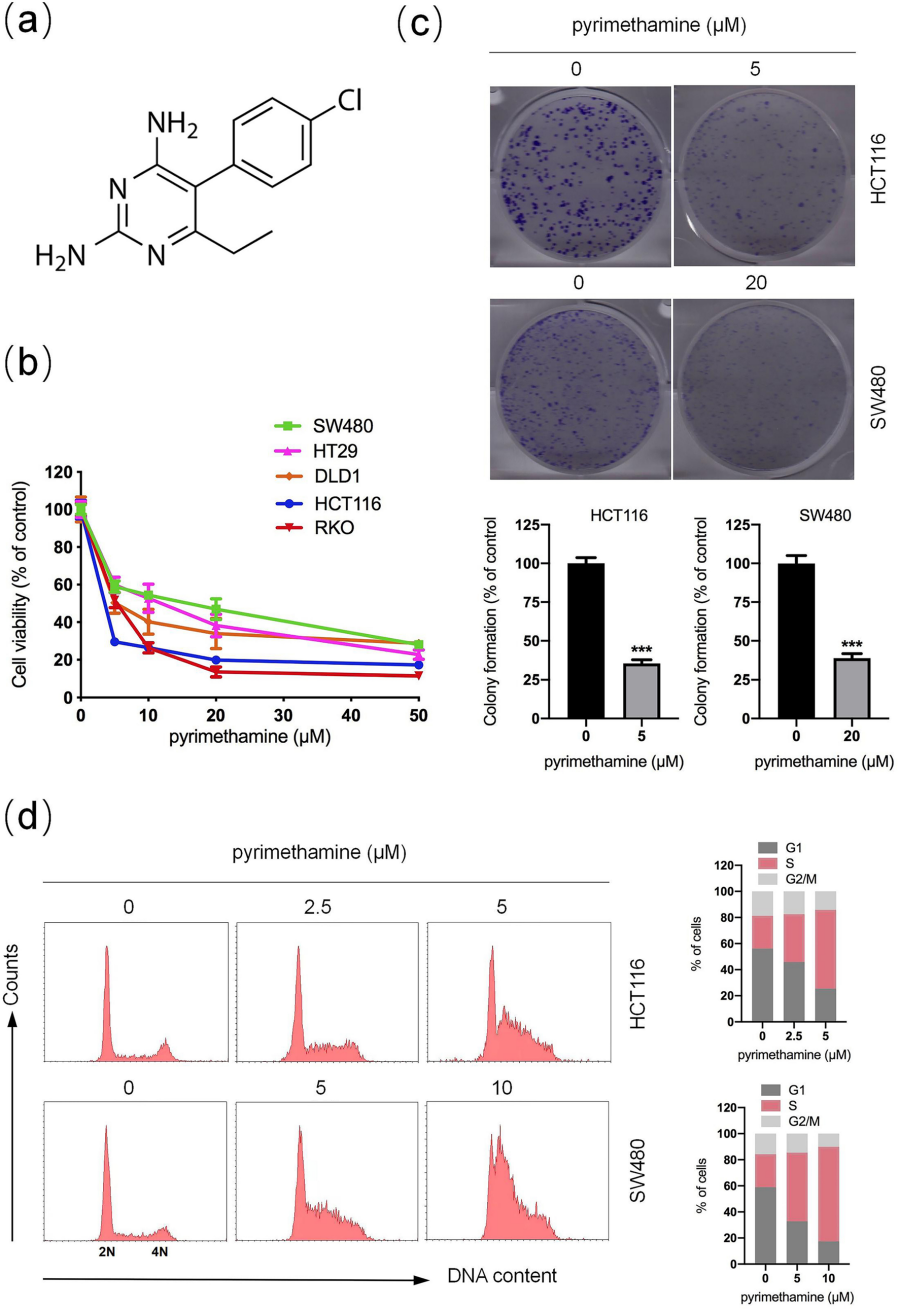
Pyrimethamine inhibits CRC cell growth. **a** The structure of pyrimethamine (Pyr). **b** Cell viability was measured using CCK-8 assays following treatment with pyrimethamine at the indicated concentrations for 72 h in HCT116, SW480, HT29, DLD1, and RKO cells. The IC50 values of each cell line were 0.4 μM, 12.3 μM, 10.0 μM, 4.4 μM, and 5.0 μM. **c** HCT116 and SW480 cells were incubated with the indicated concentrations of pyrimethamine for 8 days, and then HCT116 and SW480 cell colonies were counted. **d** HCT116 and SW480 cells were incubated with the indicated concentrations of pyrimethamine for 24 h, and cell cycle distribution was analyzed using a PI staining assay. Results are shown as mean ± SD. ^***^P < 0.001

Pyrimethamine (2,4-diamino-5-p-chlorophenyl-6-ethyl-pyrimidine; Pyr), a dihydrofolate reductase (DHFR) inhibitor, can block the synthesis of folic acid and DNA, and its structure is shown in Fig. 1a [3]. Pyrimethamine is used to treat malaria and toxoplasmosis by suppressing plasmodium and toxoplasma's proliferation and survival [3, 4]. Recently, some studies have demonstrated that pyrimethamine has an anticancer effect [5–10]. For instance, Liu et al*.* indicated that pyrimethamine could inhibit proliferation and metastasis in lung cancer cells by targeting DHFR and thymidine phosphorylase [7]. However, the effects and mechanisms of pyrimethamine on CRC have not been reported yet. In this study, our results showed that pyrimethamine inhibited the growth of CRC cells both in vitro and in vivo. The p38MAPK-p53 axis might play an important role in pyrimethamine-induced S-phase arrest followed by cellular senescence. Interestingly, we found that pyrimethamine could increase the proportion of activated CD8^+^ T-cell, which increased CD8^+^ T-cell mediated cytotoxicity to CRC cells. Our results suggested that pyrimethamine may be a potential candidate agent for CRC treatment.

## Materials and methods

### Chemical and reagents

Pyrimethamine and SB203580 (Selleck Chemicals, Houston, TX, USA) were resuspended in DMSO and stored at − 20 °C. Antibodies against p38, phospho-p38 (p-p38), and phospho-p53 (p-p53) were purchased from Cell Signaling Technology (Beverly, MA, USA). Antibodies against p53, GAPDH, anti-rabbit immunoglobulin G, and anti-mouse immunoglobulin G horseradish peroxidase-conjugated secondary antibodies were purchased from Proteintech Group (Chicago, IL, USA).

### Cell culture

The CRC cell lines SW480, HCT116, DLD1, RKO, HT29, and CT26 were acquired from the Culture Collection of the Chinese Academy of Science (Shanghai, China). Cells were cultured in DMEM or RPMI 1640 containing 10% fetal bovine serum (Gibco Life Technologies, Carlsbad, CA, USA), 100 U/mL penicillin, and 10 μg/mL streptomycin (Gibco Life Technologies). All cells were grown at 37 °C in a humidified environment supplemented with 5% CO_2_.

### Cell viability assay

A Cell Counting Kit-8 (CCK-8) assay (Nanjing KeyGen Biotech Co., Ltd., Nanjing, Jiangsu, China) was used to test the effect of pyrimethamine on cell viability. Cells were seeded in 96-well plates (5000 cells per well) and treated with various concentrations of pyrimethamine for 72 h. Then, 20 μL CCK-8 solution was added to each well and incubated for another 2 h. The absorbance density was measured on a 96-well plate reader (Thermo Fisher Scientific, Waltham, MA, USA) at a wavelength of 450 nm.

### Colony formation assay

HCT116 (1 × 10^3^ cells per well) and SW480 (2 × 10^3^ cells per well) cells were cultured in 6-well plates and then treated with pyrimethamine for eight days. After incubation with pyrimethamine, cell colonies were rinsed with phosphate-buffered saline (PBS), fixed with anhydrous methanol for 5 min, and stained with crystal violet for 5 min. Images of the cell colonies were obtained using an Epson scanner (Suwa, Nagano, Japan).

### Cell cycle analysis

Cells were seeded in 6-well plates and then treated with pyrimethamine for 24 h. HCT116 and SW480 cells were harvested, rinsed with PBS, and fixed with 70% ethanol in 15-ml centrifuge tubes overnight at 4 °C. Then, the cells were washed and stained with propidium iodide (PI) (BD Biosciences, Franklin Lake, NJ, USA) at RT for 10 min. Cell cycle distribution was analyzed by flow cytometry using CytoFLEX (Beckman Coulter, CA, USA).

### Apoptosis assay

Cell apoptosis analysis was estimated using an Annexin V-FITC/PI dual staining kit (Nanjing KeyGen Biotech Co., Ltd.). Briefly, cells were cultured in 6-well plates and incubated with pyrimethamine for 96 h. Then, the cells were collected and suspended in binding buffer with PI and Annexin V-FITC at RT for 15 min. Cell apoptosis rates were finally detected by flow cytometry. Annexin V-FITC^+^ and annexin V-FITC^+^-PI^+^ cells were considered apoptotic cells.

### SA-β-gal assay

Cells were grown in 6-well plates and treated with pyrimethamine (5 μM, 10 μM and 20 μM) for 96 h. The Senescence-associated β-galactosidase (SA-β-gal) Staining Kit (Beyotime, Shanghai, China) was used to detect SA-β-gal activity. The staining procedures were performed according to the manufacturer's instructions. Finally, we observed and photographed the stained cells under a microscope (Olympus, Tokyo, Japan).

### Real-time quantitative PCR (RT–PCR) analysis

Total RNA was extracted using the RNA-Quick Purification Kit (Shanghai Yishan Biotechnology Co., Ltd, Shanghai, China) and converted into cDNA using PrimeScript RT Master Mix (TaKaRa, Dalian, Liaoning, China). Quantitative RT–PCR was carried out using the SYBR Green Premix Ex Taq II kit (TaKaRa). The primers used were as follows:

human IL-6 forward: 5′-ACTCACCTCTTCAGAACGAATTG-3′;

human IL-6 reverse: 5′-CCATCTTTGGAAGGTTCAGGTTG-3′;

human CCL2 forward: 5′-CCATGGACCACCTGGACAAGCA-3′;

human CCL2 reverse: 5′-GGTGTCTGGGGAAAGCTAGGGG-3′;

human CXCL1 forward: 5′-AGGGAATTCACCCCAAGAAC-3′;

human CXCL1 reverse: 5′-TAACTATGGGGGATGCAGGA-3′;

human CXCL10 forward: 5′-GTGGCATTCAAGGAGTACCTC-3′;

human CXCL10 reverse: 5′-TGATGGCCTTCGATTCTGGATT-3′;

human GAPDH forward: 5′-GCACCGTCAAGGCTGAGAAC-3′;

human GAPDH reverse: 5′-TGGTGAAGACGCCAGTGGA-3′;

mouse IFNγ forward: 5′-GCCACGGCACAGTCATTGA-3′;

mouse IFNγ reverse: 5′-TGCTGATGGCCTGATTGTCTT-3′;

mouse TNFα forward: 5′-CAGGCGGTGCCTATGTCTC-3′;

mouse TNFα reverse: 5′-CGATCACCCCGAAGTTCAGTAG-3′;

mouse GAPDH forward: 5′-TGACCTCAACTACATGGTCTACA-3′;

mouse GAPDH reverse: 5′-CTTCCCATTCTCGGCCTTG-3′;

All values were normalized by GAPDH expression. The 2^−ΔΔCT^ method was used to analyse the mRNA expression of target genes.

### RNA-seq analysis

Total RNA from HCT116 cells treated with pyrimethamine at 0 μM or 20 μM was extracted using the trizol method. NovelBio performed the RNA-seq analyses. Illumina Novaseq 6000 was used to perform RNA-seq. Differentially expressed genes (DEGs), which were defined as ± one fold change and false discovery rate (FDR) < 0.01, were calculated by RNA-seq by expectation maximization and passion distribution. DEGs were then analyzed for enriched gene pathways using KEGG pathway analysis (HTTP:// www.genome.jp/kegg/pathway.html).

### Western blot analysis

RIPA buffer (Cell Signaling Technology) with phosphatase and protease inhibitors (Nanjing KeyGen Biotech Co., Ltd.) was utilized to lyse cells. A BCA protein quantitation kit (Thermo Fisher Scientific, Waltham, MA, USA) was used to determine protein concentrations. The exact amount of protein samples was separated by SDS–PAGE and transferred to a polyvinylidene fluoride membrane. Then, the membrane was blocked with 5% nonfat dry milk for 2 h, and incubated with primary antibodies overnight at 4 °C. The next day, after washing three times with TBST buffer, the membranes were incubated with secondary antibodies at RT for 1 h. Finally, enhanced chemiluminescence detection reagents (Santa Cruz Biotechnology, CA, USA) were used to observe the protein bands.

### Animal study

We used five-week-old male BALB/c mice to establish the CT-26 xenograft model. CT-26 (1 × 10^5^) cells were injected subcutaneously into the right flanks of mice. The mice were randomly assigned to two groups seven days after being injected: the control group (0.5% sodium carboxymethylcellulose, i.g.) and the pyrimethamine treatment group (60 mg/kg/d pyrimethamine i.g.). Tumor sizes were measured every other day. Moreover, tumor volumes were calculated using the following formula: a^2^ × b/2, where a is the minor diameter and b is the diameter perpendicular to a. Finally, mice were humanely sacrificed at day 15, then tumors were removed, weighed and used for further studies. A part of the tumor was embedded in paraffin and sectioned to detect CD8 and Ki67 by immunohistochemical analysis. The remaining tumor tissue was used to assess IFNγ and TNFα mRNA expression by RT–PCR. All animal studies were conducted according to the guidelines of the Committee on the Ethics of Animal Experiments of the Sixth Affiliated Hospital, Sun Yat-sen University.

### Statistical analysis

The statistical analysis was performed with GraphPad Prism 6.02. Unpaired Student’s t tests were employed between study groups. Differences at *p* < 0.05 were considered statistically significant.

## Results

### Pyrimethamine suppresses the growth of CRC cells

We first examined the anticancer effects of pyrimethamine on various CRC cell lines. HCT116, DLD1, RKO, SW480, and HT29 were treated with various concentrations of pyrimethamine for 72 h, and then their cell viabilities were measured using a CCK8 assay. As shown in Fig. 1b, pyrimethamine significantly inhibited the growth of CRC cells in a dose-dependent manner. Moreover, the colony formation assay showed that pyrimethamine effectively inhibited the clonogenic growth of HCT116 and SW480 cells (Fig. 1c). To evaluate whether the pyrimethamine-induced inhibition of cell growth was related to cell cycle arrest, the effect of pyrimethamine on cell cycle distribution was analyzed by flow cytometry. Figure 1d shows that the number of cells in S-phase was markedly increased in pyrimethamine-treated HCT116 and SW480 cells compared with control cells. These data revealed that pyrimethamine might inhibit cell growth by inducing S-phase arrest in CRC cells.

### Pyrimethamine induces cellular senescence rather than apoptosis in CRC cells

**Fig. 2:**
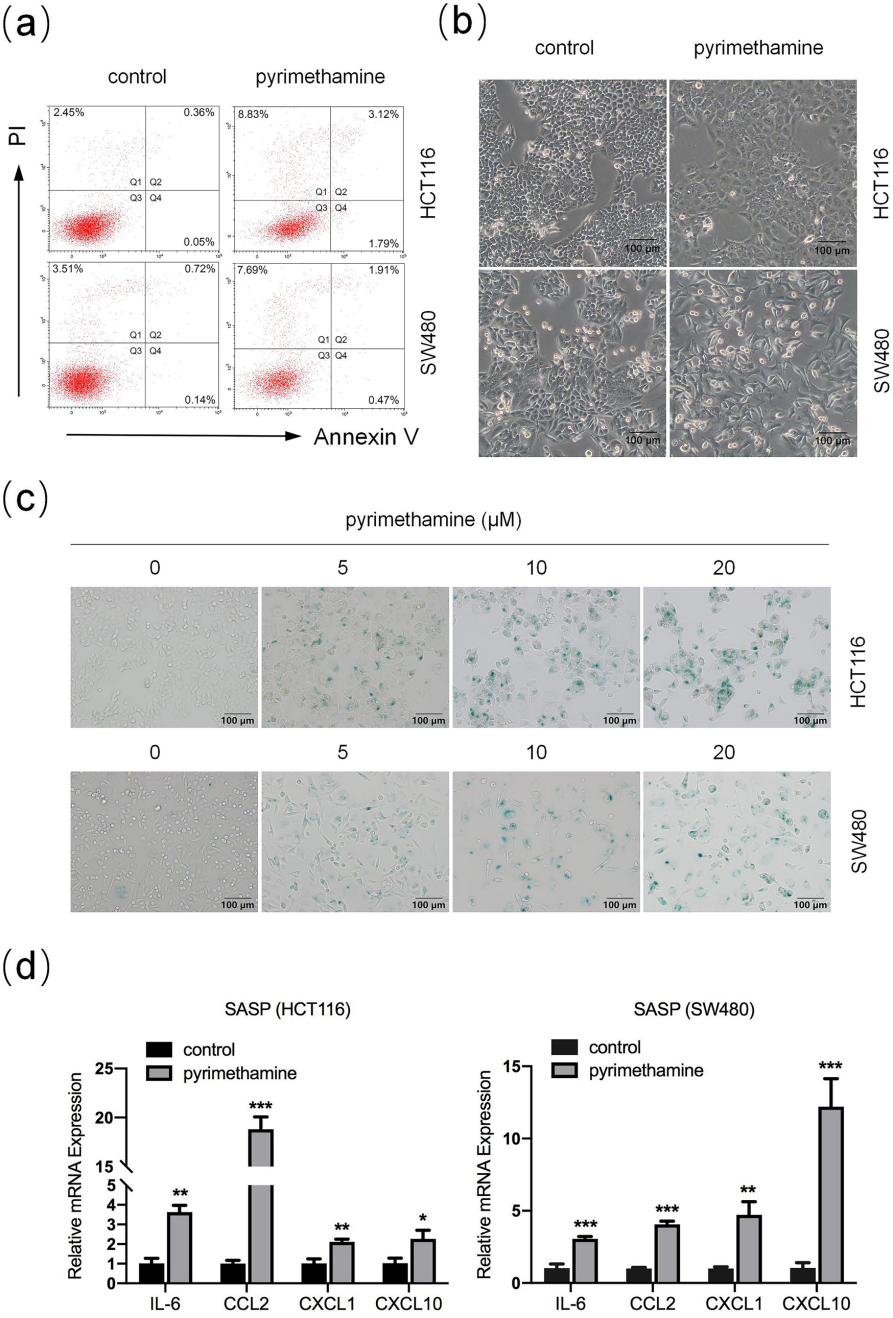
Pyrimethamine induces cellular senescence rather than apoptosis in CRC cells. **a** HCT116 and SW480 cells were exposed to the indicated concentrations of pyrimethamine for 96 h, and apoptosis was determined by flow cytometry. **b** The enlarged and flattened morphology was visualized under a light microscope after treatment with pyrimethamine (20 μM) for 48 h. scale bar = 100 μm. **c** SA-β-Gal staining was performed in HCT116 and SW480 cells treated with pyrimethamine for 96 h. Scale bar = 100 μm. **d** Changes in relative mRNA expression levels of IL-6, CCL2, CXCL1, and CXCL10 in HCT116 and SW480 cells treated with or without pyrimethamine. Results are shown as mean ± SD. *P < 0.05, **P < 0.01, ***P < 0.001

Previous reports have shown that pyrimethamine can kill cancer cells through apoptosis [6–8]. Nevertheless, our results showed that treatment with pyrimethamine at the high concentration (20 μM) and for a long period (96 h) still did not lead to an increased percentage of apoptosis in either HCT116 or SW480 cells (Fig. 2a). However, after pyrimethamine treatment cells displayed an enlarged and flattened morphology (Fig. 2b), which might herald cellular senescence [11]. Thus, we focused on whether senescence was related to pyrimethamine-mediated cell growth inhibition. HCT116 and SW480 were treated with pyrimethamine at the indicated concentrations for 96 h and stained with SA-β-gal. As shown in Fig. 2c, pyrimethamine treatment markedly upregulated the percentages of SA-β-gal-positive cells. Senescent cells present a senescence-associated secretory phenotype (SASP) characterized by the increased expression of cytokines and chemokines such as IL-6, CCL2, CXCL1, and CXCL10 [12, 13]. As shown in Fig. 2d, the RT–PCR results showed that pyrimethamine increased the expression of IL-6, CCL2, CXCL1, and CXCL10 in HCT116 and SW480 cells. The above results indicated that pyrimethamine inhibits the growth of CRC cells through induction of S-phase arrest followed by cellular senescence.

### The p38MAPK-p53 axis is probably involved in pyrimethamine-induced senescence

**Fig. 3:**
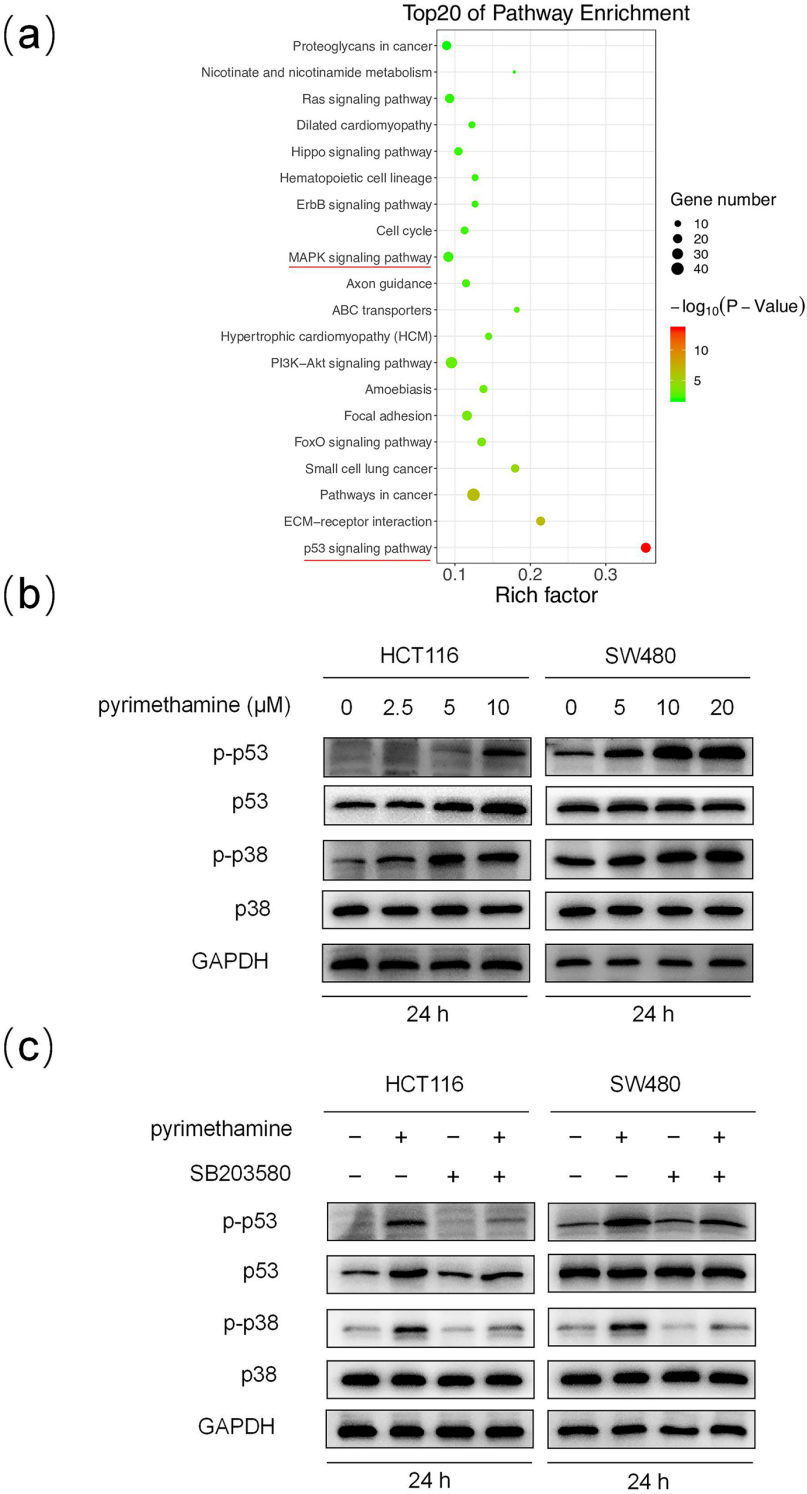
The activation of p38MAPK-p53 might be involved in pyrimethamine-induced senescence. **a** KEGG pathway enrichment of DEGs. **b** HCT116 and SW480 cells were treated with pyrimethamine for 24 h, and western blot assays detected the expression of p38, p-p38, p53, and p-p53. **c** Immunoblot analysis of p38, p-p38, p53, and p-p53 in cells treated with pyrimethamine for 24 h in the presence and absence of SB203580

To clarify the mechanism underlying pyrimethamine-induced senescence, we used RNA-seq analysis to investigate the key pathways. The top 20 most significantly enriched pathways are shown in Fig. 3a. It seemed that the p53 signaling pathway was the most notably enriched. Many reports have shown that the p53 signaling pathway is closely related to cellular senescence [14]. We next selected the p53 signaling pathway for further study. As shown in Fig. 3b, the western blot results showed that the expression levels of p53 and p-p53 in HCT116, and p-p53 in SW480 were significantly increased following pyrimethamine treatment, indicating that pyrimethamine could activate the p53 pathway in CRC. Then, we explored how pyrimethamine activated the p53 pathway. It has been reported that p38MAPK activation can lead to the phosphorylation of p53 and upregulate p53-dependent transcription [15]. Moreover, the MAPK signaling pathway was one of the most enriched pathways in our RNA-seq results (Fig. 3a). Therefore, we detected whether the activation of p38MAPK was involved in p53 pathway activation. Pyrimethamine markedly increased the phosphorylation level of p38MAPK, whereas the total p38MAPK expression level was not affected (Fig. 3b) in CRC cells, indicating that the p38MAPK pathway was activated by pyrimethamine. Interestingly, when p38MAPK activation was blocked by the p38MAPK inhibitor SB203580, pyrimethamine-induced activation of p53 was partly abrogated in CRC cells (Fig. 3c). Taken together, these results demonstrated that pyrimethamine activates p38MAPK-p53 axis and this pathway is probably involved in the induction of senescence [16–18].

### Pyrimethamine inhibits tumor growth in vivo

**Fig. 4:**
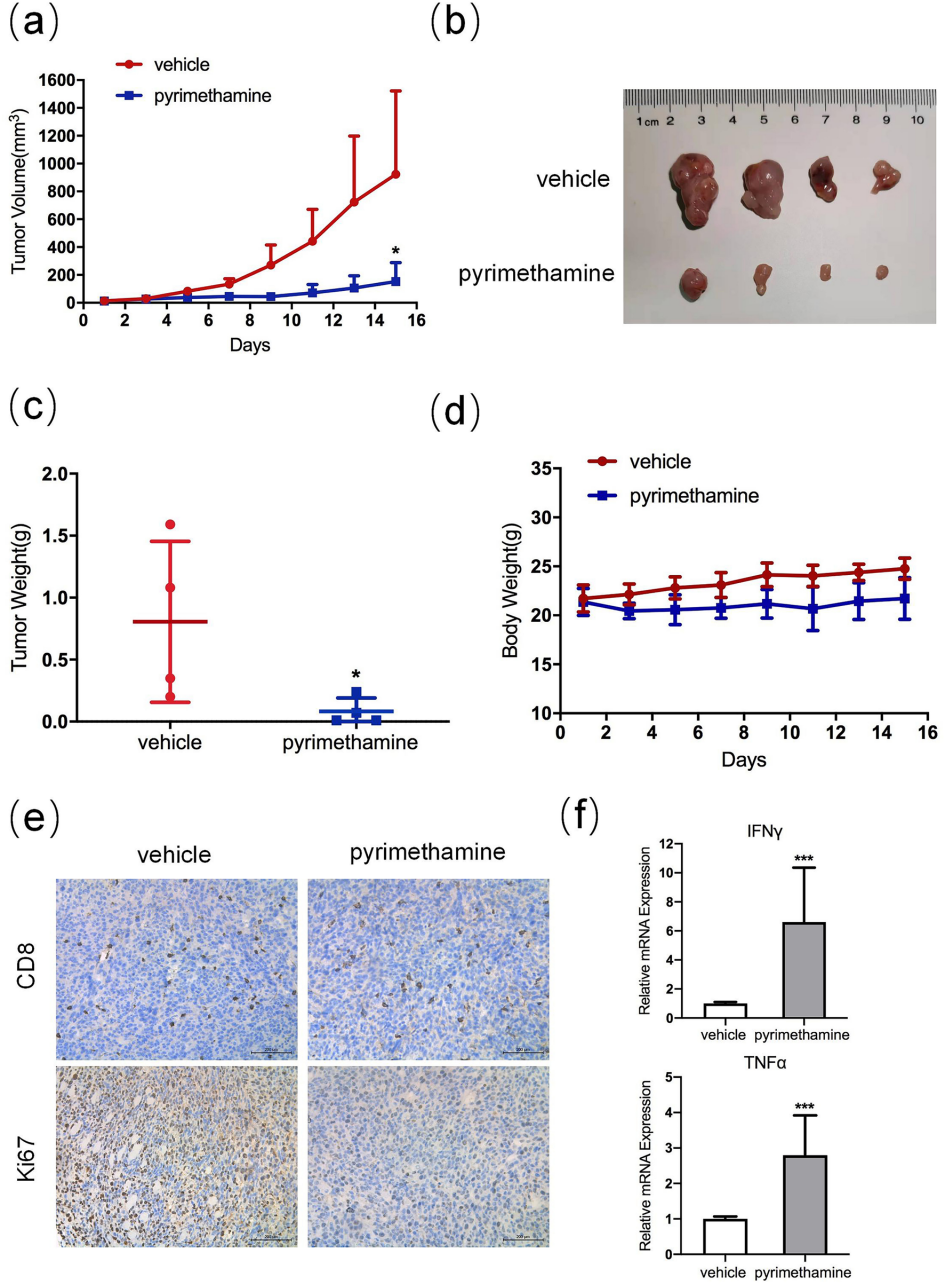
Pyrimethamine suppresses CRC progression in vivo. Mice were subcutaneously inoculated with CT26 and then treated with vehicle or pyrimethamine (60 mg/kg/day). **a** Changes in tumor volume during the experiment. **b** Tumor images at the end of the experiment. **c** Tumor weight of subcutaneous xenografts in mice. **d** Changes in mouse weights during the experiment. **e** Immunohistochemical staining for CD8 and Ki67 in sections of mouse tumor tissues from the control group and the pyrimethamine-treated group. Scale bar = 200 μm. **f** RT–PCR results of IFNγ and TNFα in subcutaneous tumor tissues from the control group and the pyrimethamine-treated group. Results are shown as mean ± SD. *P < 0.05, ***P < 0.001

To further determine whether pyrimethamine restrained the growth of CRC cells in vivo, we subcutaneously injected CT26 cells into BALB/c mice. The tumors from the pyrimethamine-treated group showed decreased volume and weight compared with tumors from the control group (Fig. 4a–c). Furthermore, the Ki-67 index was lower in the pyrimethamine treatment group, indicating that pyrimethamine could inhibit CRC cell proliferation in vivo (Fig. 4e). Moreover, the mouse body weights between the two groups were not significantly different, suggesting that pyrimethamine produced no major side effects in vivo (Fig. 4d). Previous reports have indicated that pyrimethamine could activate CD8^+^ T lymphocytes in breast cancer, and some reports have shown that restoring p53 activity can enhance CD8^+^ T-cell antitumor immunity [19, 20]. We therefore, assessed the impact of pyrimethamine on CD8^+^ T cells in CRC. As shown in Fig. 4e, f, although the numbers of total CD8^+^ T cells remained unchanged, the transcription levels of cytokines IFNγ and TNFα were notably increased in the pyrimethamine group, indicating that CD8^+^ T cells were activated in the tumor microenvironment after pyrimethamine treatment (Fig. 4e, f). All these results suggested that pyrimethamine not only impedes the growth of CRC directly but also boosts CD8^+^ T-cell mediated cytotoxicity.

## Discussion

CRC is one of the most lethal malignancies globally, and the current treatment agents for CRC are not very satisfactory [1, 21]. Thus, the development of novel therapeutic drugs with higher efficacy in treating CRC is urgently needed. This study demonstrated that pyrimethamine could inhibit cell growth by inducing S-phase arrest followed by cellular senescence. Furthermore, the p38MAPK dependent activation of p53 was involved in these effects. Moreover, our in vivo results showed that pyrimethamine could activate CD8^+^T cells. To our knowledge, this is the first report to show that pyrimethamine can induce growth inhibition in CRC cells both in vitro and in vivo, which demonstrates the potential of pyrimethamine as a candidate for CRC treatment.

DHFR is a critical enzyme for synthesizing folic acid and DNA [22]. As known, tumor cells divide more rapidly compared with normal cells [23], indicating that DHFR could be an effective target for killing cancer cells. Pyrimethamine is a DHFR inhibitor, and some recent studies have shown that pyrimethamine has inhibitory effects on cancer cells [5–8, 24, 25]. Zhou et al*.* demonstrated that pyrimethamine hindered cell cycle progression by arresting cancer cells in S-phase and suppressing cell proliferation in prostate cancer [8]. However, the pharmacological effects and mechanisms of pyrimethamine in CRC have not been reported. In this study, our results revealed that pyrimethamine could inhibit growth and induce S-phase cell cycle arrest in CRC cells, consistent with previous prostate cancer results. Moreover, in melanoma, the results from Tommasino C et al*.* revealed that pyrimethamine could induce apoptotic cell death [25]. However, our results verified that treatment with pyrimethamine at the high concentration (20 μM) and for a long period (96 h) could not induce cellular apoptosis in CRC cells. After long-term cell cycle arrest, one of the outcomes of cancer cells is senescence [26]. Our results showed that pyrimethamine could inhibit CRC cell growth through cellular senescence rather than apoptosis in CRC cells in vitro. These results in vitro suggested that pyrimethamine can be an effective anti-CRC agent.

p53 is a well-known tumor suppressor as the activation of p53 can cause cell cycle arrest or cellular senescence in cancer cells [27, 28]. Nevertheless, loss of p53 function has been found in over half of CRC [29]. Therefore, reactivating p53 seems to be a valid therapeutic strategy in treating CRC. Our RNA-seq results showed that the p53 signaling pathway was significantly enriched after CRC cells were treated with pyrimethamine. Moreover, the western blot results demonstrated that pyrimethamine could activate the p53 pathway in CRC cells. The p53 signaling pathway was previously shown to be closely related to cellular senescence, and anticancer agents such as resveratrol and curcumin could cause p53-dependent senescence in osteosarcoma and cervical cancer [30, 31]. Similarly, our results indicated that pyrimethamine might induce senescence via activating the p53 pathway in CRC cells.

But how does pyrimethamine activate p53 in CRC cells? Activating p53 by p38MAPK in colorectal cancer was previously reported [32]. MAPK pathway plays an essential role in cancer development. ERKs, JNKs, and p38MAPKs are the three prominent MAPK family members in mammalian cells, among which p38MAPK is well known to be closely linked to cellular senescence [33–36]. Surprisingly, the MAPK signaling pathway was one of the most enriched pathways in our RNA-seq results. The western blot results revealed that pyrimethamine could activate the p38MAPK pathway in CRC cells. Moreover, activation of p53 was partially restored in pyrimethamine-treated CRC cells when they were co-treated with the p38MAPK inhibitor SB203580. These results suggested that pyrimethamine might mediate the activation of p53 followed by cellular ageing via activating p38MAPK.

CD8^+^ T-cell are essential mediators of a protective immune response in cancers. Activated CD8^+^ T-cell exert their cytotoxic function through direct cytotoxicity and by producing cytokines such as IFNγ and TNFα [37, 38]. It has been reported that pyrimethamine can decrease the immunosuppressive microenvironment and enhance CD8^+^ T-cell function by increasing the release of cytotoxic granules in breast cancer [5]. Similarly, in this study, the proportion of activated CD8^+^ T-cell was increased after pyrimethamine treatment, suggesting that pyrimethamine may inhibit tumor growth by activating CD8^+^ T-cell. However, we only detected cytokines related to CD8^+^ T-cell activation, and further experiments are still needed to clarify the mechanism of pyrimethamine-mediated CD8^+^ T-cell activation.

## Conclusions

**Fig. 5:**
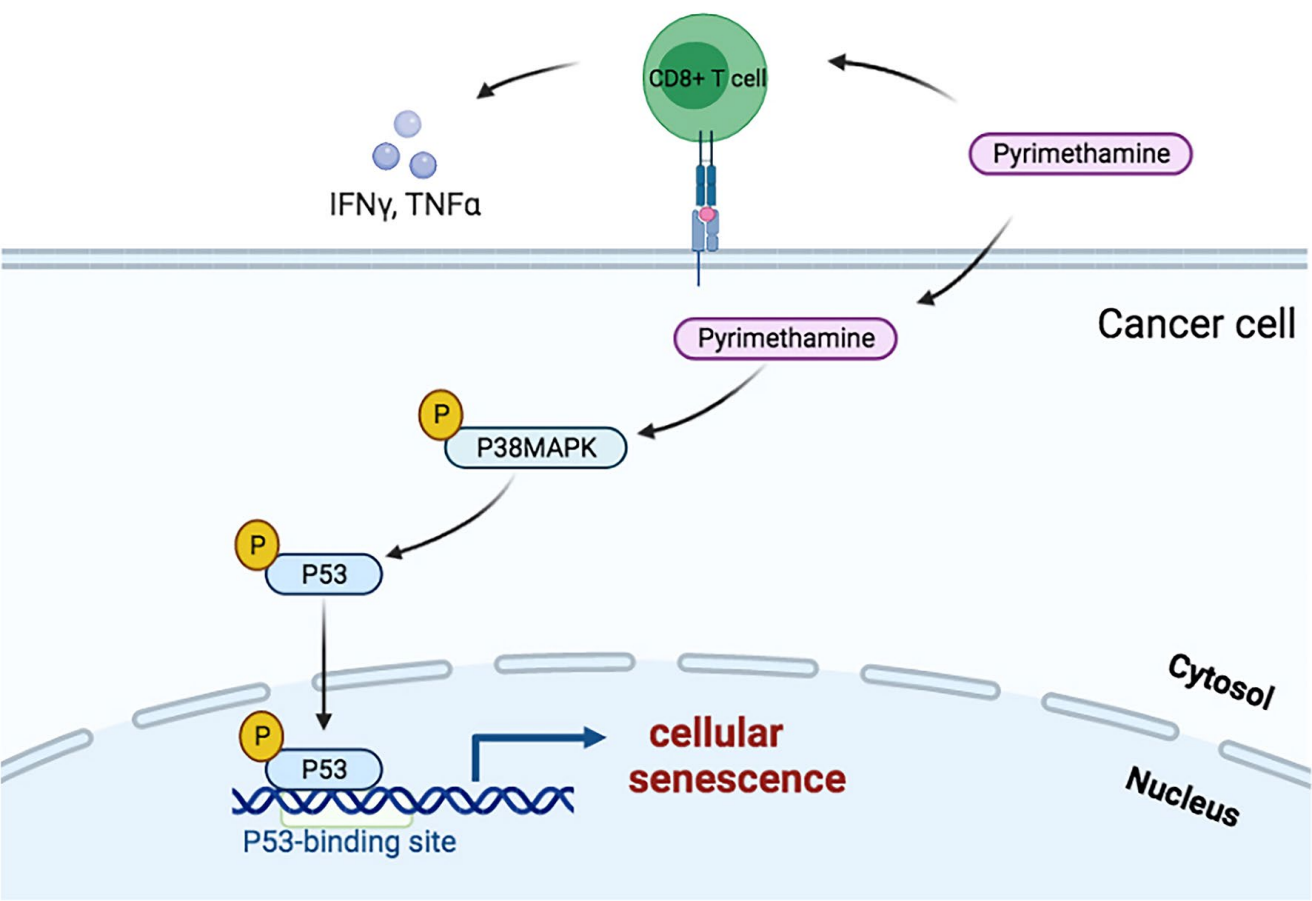
Schematic diagram of the anti-CRC effects of pyrimethamine on CRC cells. (Drawn by using BioRender (https://app.biorender.com/))

In summary, our data demonstrated that the p38MAPK-p53 axis might play an important role in pyrimethamine-induced S-phase arrest followed by cellular senescence in vitro. Furthermore, our findings also revealed that pyrimethamine boosted CD8^+^ T-cell mediated cytotoxicity and showed antitumor effects in vivo (Fig. 5). In conclusion, all of our results indicated that pyrimethamine might be a potential candidate agent for CRC treatment.
